# Respiratory muscle training during mechanical ventilation: a systematic review

**DOI:** 10.1186/cc14311

**Published:** 2015-03-16

**Authors:** D Brace, M Parrotto, C Urrea, A Goffi, A Murray, E Fan, L Brochard, N Ferguson, E Goligher

**Affiliations:** 1 UHN, Toronto, ON, Canada

## Introduction

Respiratory muscle weakness is common in mechanically ventilated patients and impairs liberation from ventilation. Inspiratory muscle training (IMT) might accelerate liberation from mechanical ventilation. We undertook to summarize previously published IMT protocols and the impact of IMT on respiratory muscle function and clinical outcomes.

## Methods

We searched multiple databases using a sensitive search strategy combining MeSH headings and keywords for studies of IMT during MV. Studies were adjudicated for inclusion and data were abstracted independently and in duplicate. Methodological quality was assessed using the GRADE system.

## Results

**Figure 1:**
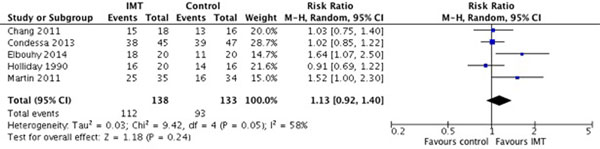
**Effect of IMT on the rate of successful weaning from mechanical ventilation**.

Eleven studies met the inclusion criteria; of these, six were randomized controlled trials and five were observational studies. A variety of IMT techniques were employed including inspiratory threshold loading (eight studies), biofeedback to increase inspiratory effort (one study), chair-sitting (one study) and diaphragmatic breathing pattern training (one study). Threshold loading was achieved by application of an external device (six studies) or increases in the inspiratory pressure trigger setting (two studies). Most studies implemented IMT in the weaning phase (*n *= 5) or after difficult weaning (*n *= 5); one study implemented IMT within 24 hours of intubation. IMT was associated with greater increases in maximal inspiratory pressure compared with control (six studies, mean difference 7.6 cmH_2_O (95% CI 5.8, 9.3), *I*^2 ^= 0%). There were no significant differences in the duration of MV (six studies, mean difference -1.1 days (95% CI -2.5, 0.3), *I*^2 ^= 71%) or the rate of successful weaning (Figure 1; five studies, risk ratio 1.13 (95% CI 0.92, 1.40), *I*^2 ^= 58%). The GRADE quality of evidence was low for all these outcomes; risk of bias was high for most studies and summary effects were imprecise and inconsistent. No serious adverse events related to IMT were reported.

## Conclusion

IMT in mechanically ventilated patients appears safe and well-tolerated and improves respiratory muscle function. IMT was not associated with accelerated liberation from mechanical ventilation. However, because the included studies had important methodological limitations and employed varying methods of IMT, we cannot draw firm conclusions about the effect of IMT on clinical outcomes.

